# MAOA uVNTR Polymorphism Influence on Older Adults Diagnosed with Diabetes Mellitus/Systemic Arterial Hypertension

**DOI:** 10.1155/2023/8538027

**Published:** 2023-07-25

**Authors:** Gabriel Moura Alves Seixas, Renata de Souza Freitas, Caroline Ferreira Fratelli, Calliandra Maria de Souza Silva, Luciano Ramos de Lima, Marina Morato Stival, Silvana Schwerz Funghetto, Izabel Cristina Rodrigues da Silva

**Affiliations:** ^1^Graduate Program in Health Sciences and Technologies, Faculty of Ceilândia, University of Brasilia, Federal District, Brasília, Brazil; ^2^University Center of Brasília (UniCEUB), Brasília, Brazil; ^3^Graduate Program in Genomic Sciences and Biotechnology, Catholic University of Brasília, Brasília, Brazil; ^4^Faculty of Ceilândia, University of Brasilia, Federal District, Brasília, Brazil

## Abstract

**Background:**

Monoamine oxidase (MAO) is involved in several biological processes associated with well-being and mental health, and alterations in its function might directly impact various mental disorders. Some mental disorders concomitantly occur in individuals with clinical characteristics, such as substance abuse and diabetes.

**Objective:**

To analyze the functional MAOA uVNTR polymorphism genotype frequency in an older adult population with diabetes mellitus/arterial hypertension and associate this frequency with clinical characteristics impacting daily life. *Methodology*. Older adults diagnosed with diabetes mellitus, systemic arterial hypertension, or both (DM/SAH) were selected and had their MAOA gene genotyped for uVNTR polymorphism. The revised Beck Depression Inventory (BDI) and a questionnaire were also applied to determine their mental health and clinical characteristics.

**Results:**

The allelic variants detected among the participants were the 2R, 3R, 4R, and 3R/4R heterozygous genotypes. Genotypes solely containing the 3R allele had patients who marked yes for smoking and alcoholism, and only those with the 3R genotypes (female 3R/3R homozygote or male 3R^*∗*^ hemizygote) were significant. Although not statistically significant, only 3R and 3R/4R genotypes presented cases of severe depression per the revised BDI interpretations.

**Conclusion:**

The MAOA uVNTR polymorphism's low-activity 3R allele presence in an older adult population diagnosed with DM/SAH may represent a risk for developing substance use (alcohol and smoking) dependence.

## 1. Introduction

The rate of population aging is increasing [1], transforming the profiles of existing diseases' incidence and prevalence. Older adults, for example, are more likely to develop noncommunicable chronic diseases (NCDs) at the expense of infectious diseases. Although not having contagious elements [2], NCDs are the leading cause of worldwide death [3]. NCDs include cancer, diabetes, cardiovascular, and respiratory diseases [4, 5]. Some NCDs occur concomitantly with depressive and anxiety disorders, and with the increase in mental disorders cases, taking care of mental health and well-being has become a target for NCD management and reduction of these comorbidities [4].

People with mental disorders, especially depression, contribute significantly to the global burden of disease [6, 7] and are a significant cause of reduced life expectancy [8]. These compounding problems alert the public health system to the need for prevention and treatment protocols, especially as geriatric depression has a more chronic and persistent characteristic than younger patients, becoming disabling and compromising functional aging [9].

With the COVID-19 pandemic and the upsurge in mental disorders, such as anxiety and depression, some aspects of senescence and mental health met with questions about how to mitigate the mental vulnerabilities of older adults to promote functional aging, in which physical and mental well-being is preserved [4, 10, 11]. The pandemic also presented variations in the risk of getting involved with or worsening an addictive substance use, such as alcohol among others, as a maladaptive coping strategy, depending on alcohol availability, social distancing, changes in daily routine, income reduction, and sometimes due to the reduced or modified mental health and addiction treatment services provided during the period [12]. However, “hasty and untimely generalizations” based on the previous socioeconomic crises should be avoided [12].

In the literature, specific genes and enzymes linked to the central nervous system have been proven to affect the etiological process of mental disorders [13] and substance abuse-induced dysregulation of neurotransmissions [14]. For instance, both mental disorders and abused substances, directly or indirectly, are altered by monoaminergic (both dopaminergic and serotonergic) pathways [14–16]. Monoamine oxidase (MAO), found in the outer membrane of the mitochondria, can metabolize neurotransmitters, including serotonin, norepinephrine, adrenaline, and dopamine, therefore, being paramount in the modulatory processes of the central and peripheral nervous system [17]. MAO protein is present in two isoenzymes, MAO-A and MAO-B, that differ in the specificity of the neurotransmitters they metabolize [18].

The literature seeks to understand MAO's etiological role and impact on mental disorders, including how the MAOA gene's genetic and epigenetic process influences the individual [19]. Several studies indicate that MAO-A is one of the most relevant targets in neurological disorders [20]. For instance, an MAOA mutation causes Brunner's syndrome by altering its protein's functionality [21]. These associations make MAO-A study a therapeutic focus for various mental disorders [22–24]. MAO enzymes have also been studied regarding susceptibility to drug use [25, 26]. Genes' relationship with mental disorders, such as depression and substance abuse disorder, has been increasingly studied to clarify how genetics and environment impact individuals and how it influences disorders' appearance and prognosis [23, 27, 28].

The MAO-A enzyme is encoded in the MAOA gene located on the short arm of the X chromosome between the Xp11.23 and Xp11.4 bands [29]. Studies point to a functional polymorphism 1.2 kb upstream variable number of tandem repeats (uVNTR) of the gene transcription initiation site, directly interfering with its enzyme and transcriptional activity but not its gene methylation nor its mRNA abundance [29–31]. This polymorphism contains up to six copies with 30 bp repeats, in which alleles with 3R (3 repeats) is known as “low-activity” allele, while the 3.5R (3.5 repeats) and 4R (4 repeats) alleles are known as “high-activity” alleles [30–32]. In contrast, 2R (2 repeats), 5R (5 repeats), or 6R (6 repeats) are considered by some as “low-activity” alleles and by others as “high-activity” alleles; this nonconformity is probably due to their rarity in the populations studied until the moment [30, 31].

Based on existing research, this study aims to verify the MAOA uVNTR polymorphism frequency in an older adult population with DM/SAH in the Federal District, Brazil, associate this frequency with social characteristics that impact this population's daily life, and investigate the polymorphism distribution according to the Beck Depression Inventory-II (BDI-II) parameters [33, 34].

## 2. Materials and Methods

### 2.1. Research Participants

The research participants were older adult volunteers who were users of the DF's Basic Health Unit (BHU) in the Federal District (DF), Brazil, diagnosed with diabetes mellitus, arterial hypertension, or both. The study population's inclusion criteria were being 60 years or older (considered older adults in Brazil's legislation), being monitored at the BHU, and being able to understand, verbalize, and answer the proposed questions. The initial sample was *N* = 40, with 19 male and 21 female participants. The sample size was calculated using the Raosfot software (Sample Size Calculator), estimating a 50% response distribution, an 8% sampling error, and a 95% confidence interval (CI) in 50 patients who attended the collection data week, and a sample size of 38 participants was reached. The sample size of 40 patients was admitted considering possible compensation for losses, and we considered the first 40 participants who agreed to participate in the survey.

The Federal District's Health Department Research Ethics Committee approved the present study (protocol 1.355.211). All procedures were performed with the participants' understanding and written consent, following the National Research Council principles under the Brazilian legislation's resolution no. 46 (2012) and the Helsinki Declaration. After obtaining their written consent form, the participants' medical information and biological material were collected.

### 2.2. Sample Collections

Approximately 15 mL of venous blood was collected after each patient had fasted for 12 hours. The second stage consisted of applying a structured questionnaire to assess the patient's demographic profile, composed of closed questions regarding gender, age, education, lifestyle (smoking), and clinical conditions (hypertension or diabetes mellitus diagnosis). This stage sought to characterize the population involved, and the researchers also helped clarify any doubts in this stage.

### 2.3. Beck Depression Inventory-II

The Beck Depression Inventory-II (BDI-II), a multiple-choice self-report with 21 questions, was used to measure depression symptoms, with each item rated on a scale from 0 to 3. The total score was calculated by adding the 21 items' scores and ranged from 0 and 63 points, with higher scores indicating greater severity of symptoms [33]. The revised BDI-II interpretation is as follows: minimum range = 0–9, mild depression = 10–16, moderate depression = 17–29, and severe depression = 30–63 [33, 34].

### 2.4. DNA Extraction

Genomic DNA was extracted from approximately 5.0 mL of peripheral blood from each patient using the Invitrogen company's PureLink® Genomic DNA Mini Kit (catalog #k1820-02, lot #19339891), according to the manufacturer's protocol. An electrophoretic run on a 2% agarose gel stained with ethidium bromide determined the DNA integrity. Subsequently, the DNA was stored at −70°C until further use.

### 2.5. Genotyping

The polymerase chain reaction (PCR) technique was used for genotyping the MAOA uVNTR polymorphism. Each reaction contained 4.0 *μ*L of genomic DNA at a final concentration of 2.5 ng/*μ*L; 1.9*μ*L of 10x buffer (10 mM Tris and 50 mM KCl); 0.38 *μ*L of 50 mM MgCl_2_ (Ludwig Biotec, Alvorada, Rio Grande do Sul, Brazil), 1.5 *μ*L of deoxyribonucleotide triphosphate (dNTPs: 2.5 mM; Ludwig Biotec, Alvorada, Rio Grande do Sul, Brazil); 0.2 *μ*L of Taq polymerase (Ludwig Biotec, Alvorada, Rio Grande do Sul, Brazil); 0.6 *μ*L of each of the two primers (MAOA-F: 5′-ACAGCCTCGCCGTGGAGAAG-3′ and MAOA-R: 5′GAACGGACGCTCCATTCGGA-3′, 10 *μ*M; IDT technologies); and completing with Milli-Q water to a final volume of 25 *μ*L.

The PCR reaction was performed in a Techne Model TC-512 thermocycler following these sequential thermocycling conditions: initial denaturation at 95°C for 5 minutes, 35 denaturation cycles at 94°C for 30 seconds, annealing at 55°C for 30 seconds, and extension at 72°C for 30 seconds. The PCR products were subjected to electrophoresis on a 3.0% agarose gel (Amresco, Ohio, USA) with a 0.5x TBE running buffer. The gels were stained with ethidium bromide and visualized using an ImageMaster VDS gel documentation system (Pharmacia Biotech, Uppsala, Sweden). The MAOA uVNTR promoter polymorphism was interpreted based on Sabol et al. (1998)'s results, in which the PCR product sizes for 2R, 3R, 4R, and 5R were 320 bp, 350 bp, 380 bp, and 410 bp, respectively.

### 2.6. Statistical Analysis

The genotype frequency of MAOA uVNTR polymorphism was analyzed for the homozygote genotypes 2R, 3R, and 4R and the heterozygote genotype 3R/4R. These genotypes were analyzed and distributed according to the patient's clinical characteristics and the revised BDI interpretations.

Pearson's chi-square test or Fisher's exact test and odds ratio (OR) determined whether the intergroup differences between the 2R, 3R, 4R, and 3R/4R variants' genotype frequency were significant (*P* value <0.05). SPSS software (version 27.0) was used for the statistical analysis.

## 3. Results

The MAOA uVNTR polymorphism's genotypic frequency distribution in older adults diagnosed with diabetes mellitus (DM), systemic arterial hypertension (SAH), or both (DM and SAH) (DM/SAH) residing in the Federal District, Brazil, is displayed in Table 1. The genotypes found in the sample were 2R^@^ (female 2R/2R homozygote or male 2R^*∗*^ hemizygote), 3R^@^ (female 3R/3R homozygote or male 3R^*∗*^ hemizygote), 4R^@^ (female 4R/4R homozygote or male 4R^*∗*^ hemizygote), and 3R/4R, with 3R^@^, 4R^@^, and 3R/4R being the most frequents. The 3.5R, 5R, and 6R alleles were undetected among the participants. The 3R and 4R alleles were most prevalent in the sample population.

None of the detected genotypes was significantly associated with DM or SAH diagnoses nor the patients' biological sex or sleep pattern (Table 2).

Interestingly, only patients with 3R^@^ genotypes marked yes for smoking and alcoholism, and in the case of smoking habit, the presence of at least one 3R allele seems to be enough for one yes, though not significant (Table 3).

Although not statistically significant, only patients with MAOA uVNTR polymorphism's 3R^@^ and 3R/4R genotypes (at least one 3R allele) presented cases of severe depression according to the revised Beck Depression Inventory-II (BDI-II) interpretations (Table 4).

## 4. Discussion

The present study detected in a sample of older adults diagnosed with diabetes mellitus (DM), systemic arterial hypertension (SAH), or both (DM and SAH) (DM/SAH) residing in the Federal District, Brazil, with the following MAOA uVNTR polymorphism's genotypes: 2R^@^ (female 2R/2R homozygote or male 2R^*∗*^ hemizygote); 3R^@^ (female 3R/3R homozygote or male 3R^*∗*^ hemizygote), 4R^@^ (female 4R/4R homozygote or male 4R^*∗*^ hemizygote), and 3R/4R (Table 1). The most frequent genotypes in the sample were 3R^@^, 4R^@^, and 3R/4R (Table 1). Notably, none of these genotypes significantly correlated with DM or SAH diagnoses nor the patients' biological sex or sleep pattern (Table 2). Furthermore, the 3.5R, 5R, and 6R alleles were undetected in this sample population.

Certain genetic factors might corroborate with specific pathologies and etiological points, such as substance use and mental disorders [35, 36]. For instance, the monoaminergic neurotransmission system directly influences the responses that the alcohol use promotes in the body [37] by producing rewarding/reinforcer effects that increase extracellular dopamine [38]. So, alterations in these systems, genetic or epigenetic, combined with environmental factors might raise the high risk of presenting these disorders [39]. None of the detected genotypes was significantly associated with diabetes mellitus or systemic arterial hypertension diagnoses nor with the patients' biological sex or sleep pattern (Table 2).

Curiously, only 3R^@^ patients marked yes for smoking and alcoholism, and the presence of at least one 3R allele seems enough for one marked yes in the case of smoking, even if not significant (Table 3). In the literature, individuals with low-activity alleles have been related in contrast to both emotional stability [40] and antisocial behavior that promotes delinquency [41].

Kaya et al.'s [42] study with the Turkish male population found that the MAOA uVNTR low-activity allele 3R was related to impulsive behavior in people with alcohol use disorder. Likewise, MAOA uVNTR low-activity alleles 2R (presumably) and 3R were (confirmed) associated with tobacco and alcohol use in male patients with episodes of physical abuse [43, 44]. Another Brazilian study also found a correlation between 3R allele alcohol dependence, an earlier alcoholism onset, comorbid drug abuse among alcoholics, and a higher number of antisocial symptoms [45]. Like in human studies, Cervera-Juanes et al.'s [46] animal study evidenced that a lower MAOA activity level influences vulnerability and alcohol dependence.

Studies have associated tobacco use with significantly reducing the MAO enzyme's capacity and activity [47] and inhibiting its functionality [48]. Likewise, exacerbated consumption of substances, such as tobacco and alcohol, has been associated with the MAOA gene's methylation in women [49].

Although not statistically significant, only patients with at least one MAOA uVNTR 3R allele presented cases of severe depression according to the revised Beck Depression Inventory-II (BDI-II) interpretations (Table 4). These results are similar to the Rivera et al. [50] and Huang et al. [51] studies, in which patients with the 3R allele and 3R/4R genotype tended to show severe depression symptoms, directly linking this polymorphism to major depressive disorder (MDD).

Our study's main limitation was the reduced number of patients. Therefore, we recommend that future studies increase the number of patients, investigate the MAO protein level, and analyze its gene epigenetics in this population. These additions might help answer questions relevant to the etiology of mental and substance use disorders.

## 5. Conclusion

Similar to other scientific findings, our study corroborates that Brazilian older adults diagnosed with diabetes mellitus, arterial hypertension, or both with the MAOA uVNTR polymorphism's low-activity allele 3R presented a higher risk of developing alcohol and smoking dependence, impacting the lives of these older adults.

## Figures and Tables

**Table 1:** The MAOA uVNTR polymorphism's genotypic frequency in older adults diagnosed with diabetes mellitus, systemic arterial hypertension, or both from the Federal District, Brazil.

		Frequencies	Percentages (%)
Variants	2R^@^ (presumably low activity)	4	10.0
	3R^@^ (low activity)	13	32.5
	4R^@^ (high activity)	15	37.5
	3R/4R (low activity/high activity)	8	20.0
	Total	40	100.0

Note. ^@^ represents the combined female homozygote and male hemizygotes; 2R^@^ (female 2R/2R homozygote or male 2R^*∗*^ hemizygote); 3R^@^ (female 3R/3R homozygote or male 3R^*∗*^ hemizygote); 4R^@^ (female 4R/4R homozygote or male 4R^*∗*^ hemizygote); and ^*∗*^represents male hemizygotes.

**Table 2:** The MAOA uVNTR polymorphism's genotypes frequency distributed according to clinical characteristics in older adults diagnosed with diabetes mellitus (DM), systemic arterial hypertension (SAH), or both from the Federal District, Brazil.

		*MAOA uVNTR* polymorphism genotypes								
		2R^@^ (presumably low activity)		3R^@^ (low activity)		4R^@^ (high activity)		3R/4R (low activity/high activity)		*P*
		*N*	%	*N*	%	*N*	%	*N*	%	
Biological sex	Female	3	12.5	6	25.0	7	29.2	8	33.3	0.051
	Male	1	6.3	7	43.8	8	50.0	0	0.0	

SAH	Yes	3	8.6	11	31.4	13	37.1	8	22.9	0.611
	No	1	20.0	2	40.0	2	40.0	0	0.0	

DM	Yes	4	11.4	12	34.3	11	31.4	8	22.9	0.192
	No	0	0.0	1	20.0	4	80.0	0	0.0	

Sleep	Normal	3	13.6	7	31.8	9	40.9	3	13.6	0.511
	Difficulty to sleep	1	7.7	5	38.5	3	23.1	4	30.8	

Note. ^@^ represents the combined female homozygote and male hemizygotes; 2R^@^ (female 2R/2R homozygote or male 2R^*∗*^ hemizygote); 3R^@^ (female 3R/3R homozygote or male 3R^*∗*^ hemizygote); 4R^@^ (female 4R/4R homozygote or male 4R^*∗*^ hemizygote); and ^*∗*^ represents male hemizygotes.

**Table 3:** The MAOA uVNTR polymorphism's genotypes frequency distributed according to the clinical characteristics (alcoholism and smoking) in older adults diagnosed with diabetes mellitus, systemic arterial hypertension, or both from the Federal District, Brazil.

Variants	Smoking				Alcoholism			
	Yes	No	*P*	OR (IC-95%)	Yes	No	*P*	OR (IC-95%)
2R^@^ (presumably low activity)	0	4	0.601	NA	0	4	0.782	NA
3R^@^ (low activity)	3	9	0.106	7.33 (0.67–80.22)	2	10	0.111	NA
4R^@^ (high activity)	0	12	0.169	NA	0	12	0.063	NA
3R/4R (low activity/high activity)	1	6	0.608	1.39 (0.12–15.81)	0	7	0.635	NA

Note. ^@^ represents the combined female homozygote and male hemizygotes; 2R^@^ (female 2R/2R homozygote or male 2R^*∗*^ hemizygote); 3R^@^ (female 3R/3R homozygote or male 3R^*∗*^ hemizygote); 4R^@^ (female 4R/4R homozygote or male 4R^*∗*^ hemizygote); and ^*∗*^ represents male hemizygotes. NA: not applicable.

**Table 4:** The MAOA uVNTR polymorphism's genotypes frequency distributed according to the revised Beck Depression Inventory-II (BDI-II) interpretations in older adults diagnosed with diabetes mellitus, systemic arterial hypertension, or both from the Federal District, Brazil.

		*MAOA* uVNTR polymorphism genotypes				
		2R^@^(presumably low activity)	3R^@^ (low activity)	4R^@^ (high activity)	3R/4R (low activity/high activity)	*P*
		%	%	%	%	
Revised BDI-II interpretations	Minimum range	0.0	7.7	0.0	12.5	0.261
	Mild	0.0	30.8	20.0	25.0	0.213
	Moderate	25.0	23.1	33.3	25.0	0.238
	Severe	75.0	38.5	46.7	37.5	0.406

Note. ^@^ represents the combined female homozygote and male hemizygotes; 2R^@^ (female 2R/2R homozygote or male 2R^*∗*^ hemizygote); 3R^@^ (female 3R/3R homozygote or male 3R^*∗*^ hemizygote); 4R^@^ (female 4R/4R homozygote or male 4R^*∗*^ hemizygote); and ^*∗*^ represents male hemizygotes.

## Data Availability

The datasets used and analyzed during the study are available from the corresponding author upon reasonable request.
